# Maternal Diabetes and Cognitive Performance in the Offspring: A Systematic Review and Meta-Analysis

**DOI:** 10.1371/journal.pone.0142583

**Published:** 2015-11-13

**Authors:** Maria Camprubi Robles, Cristina Campoy, Llenalia Garcia Fernandez, Jose M. Lopez-Pedrosa, Ricardo Rueda, Maria J. Martin

**Affiliations:** 1 Abbott Nutrition, Research and Development-University Science Park, Granada, Spain; 2 Department of Pediatrics, School of Medicine, University of Granada, Granada, Spain; 3 SEPLIN Soluciones Estadisticas S.L., Granada, Spain; 4 Abbott Nutrition, Research and Development, Granada, Spain; McMaster University, CANADA

## Abstract

**Objective:**

Diabetes during gestation is one of the most common pregnancy complications associated with adverse health effects for the mother and the child. Maternal diabetes has been proposed to negatively affect the cognitive abilities of the child, but experimental research assessing its impact is conflicting. The main aim of our study was to compare the cognitive function in children of diabetic and healthy pregnant women.

**Methods:**

A systematic review and meta-analysis was conducted through a literature search using different electronic databases from the index date to January 31, 2015. We included studies that assessed the cognitive abilities in children (up to 14 years) of diabetic and non-diabetic mothers using standardized and validated neuropsychological tests.

**Results:**

Of 7,698 references reviewed, 12 studies involving 6,140 infants met our inclusion criteria and contributed to meta-analysis. A random effect model was used to compute the standardized mean differences and 95% confidence interval (CI) were calculated. Infants (1–2 years) of diabetic mothers had significantly lower scores of mental and psychomotor development compared to control infants. The effect size for mental development was -0.41 (95% CI -0.59, -0.24; p<0.0001) and for psychomotor development was -0.31 (95% CI -0.55, -0.07; p = 0.0125) with non-significant heterogeneity. Diabetes during pregnancy could be associated with decreased intelligence quotient scores in school-age children, although studies showed significant heterogeneity.

**Conclusion:**

The association between maternal diabetes and deleterious effects on mental/psychomotor development and overall intellectual function in the offspring must be taken with caution. Results are based on observational cohorts and a direct causal influence of intrauterine hyperglycemia remains uncertain. Therefore, more trials that include larger populations are warranted to elucidate whether gestational diabetes mellitus (GDM) has a negative impact on offspring central nervous system (CNS).

## Introduction

Maternal diabetes is a result of either pre-existing diabetes in a pregnant women (Type 1 or Type 2 Diabetes Mellitus (T1/T2DM) also known as Pre-gestational Diabetes Mellitus (PGDM)), or the development of insulin resistance and subsequent high blood glucose with onset or first diagnose during pregnancy, defined as Gestational Diabetes Mellitus (GDM) [1]. Hyperglycemia is one of the most common health complications in pregnant women. In fact, the number of women with pregnancy diabetes is expected to rise as a result of increased sedentary habits and hypercaloric diets which accounts for the global burden of obesity and diabetes [2]. According to the International Diabetes Federation (IDF) 17% (21 Million) of live births in 2013 had some form of hyperglycemia in pregnancy [1]. Diabetes prevalence in U.S. childbearing age women is 9% and it is estimated that 2–5% of all pregnancies are affected with GDM [3]. A similar prevalence is found in Europe where GDM accounts for 2–6% of all pregnancies [3]. Although due to lack of uniform criteria for diagnosis it is impossible to assess actual numbers, recent statistics estimate that the majority of diabetic pregnancies correspond to GDM (~87%) while pre-existing T1DM and T2DM account for 7% and 5%, respectively [4,5].

The impact of diabetes on maternal and infant health has been extensively investigated as an example of developmental origins of disease (*early programming*). According to the Hyperglycemia and Adverse Pregnancy Outcomes (HAPO) study, the associations between altered pregnancy glycemia and adverse health outcomes in offspring are present even below diagnostic levels of diabetes [6] which raises critical health concerns about the achievement of an appropriate glucose management in pregnancy. Potential pathways linking maternal diabetes and offspring health outcomes such as adiposity, cardiometabolic health and cognitive performance have been widely reported in numerous studies (see Fraser et al. [7] for a complete review).

The fetal environment in maternal diabetes is mainly characterized by hyperglycemia, chronic hypoxia and iron deficiency, complemented with recurrent acute changes in glucose status and acidemia [8,9]. Moreover, pregnancy altered glycemia may affect fetal neurodevelopment, have a big impact on offspring cognition, and also increase the risk of suffering from mental disorders, such as Attention Deficit and Hyperactivity Disorder (ADHD) [10,11].

However, general knowledge from epidemiology cohort studies points to very different directions, and thus impaired [11,12], unaffected [13,14] and even improved [15] cognitive function have been reported in diabetes-exposed children. To shed light on this topic, several recent reviews have been published [7,16], but there is no systematic review or meta-analysis so far that has evaluated the relationship between maternal diabetes and the cognitive ability in their offspring. Therefore, we hypothesize that a diabetic pregnancy may generate an adverse intrauterine environment which drives neurodevelopment impairment of the fetus, thus inducing critical limitations on its future cognitive abilities either in infancy or in childhood. In view of the complexity of this important area of health care, we conducted a systematic review and meta-analysis to identify possible neurocognitive harms on children of diabetic mothers in comparison to those of healthy non-diabetic women.

## Methods

Our review followed the Meta-Analyses and Systematic Reviews of Observational Studies (MOOSE) guidelines [17]. The data were presented according to the recommendations of the PRISMA statement [18].

### Literature search

A comprehensive systematic literature search was conducted through 4 different electronic databases: SciFinder (covering MEDLINE, CAplus Registry, Sreact, Chemcats and Chemlist), Scopus (covering MEDLINE, EMBASE, Compedex, World textile index, Fluidex, Geobase and Biobase), The Cochrane Library and ClinicalTrials.gov from the index date to January 31, 2015 for eligible epidemiology trials. Our search strategy included key terms that are summarized as follows: “gestational diabetes”, “diabetic mother”, “diabetes pregnancy”, “insulin gestation” and “offspring psychomotor function”, “offspring cognit*”, “children cognit*”, “child* behavior”, “learning” (see S1 Text for detailed search strategy).

### Study selection

Two authors (M.C.R. and M.J.M.) reviewed titles and abstracts of identified records to exclude any clearly irrelevant study. Candidate full-text articles were read by the same authors independently to determine whether they met inclusion criteria. When required, discrepancies were resolved by consensus with a third author (J.M.L.-P.). The literature search included articles written in English, German, French, and Spanish. The corresponding author of 2 published studies was contacted since additional information related to study design or participant recruitment was required.

Criteria for selecting population were restricted to studies which included offspring of mothers with PGDM or GDM with an age up to 14 years and with any length of follow-up. Studies recruiting infants or children from all demographic and geographic settings were eligible. In most countries, diabetes during pregnancy is controlled according to international guidelines; however, the type of diabetes control was not considered as a key criterion for inclusion since this meta-analysis was not conducted to evaluate different methods of glycemic control.

### Outcomes of interest

Primary outcomes were defined as follows: (a) Mental Development Index (MDI) in Bayley Scale of Infant Development, (BSID) [19,20], (b) Motor function measured by Bayley Psychomotor Development Index (PDI) [19,20], and (c) Intelligence Quotient (IQ) yielded by different Wechsler scales [21,22] or the Standford-Binet Intelligence Scale [23]. As a secondary outcome we defined the language development examined through specific communicative and vocabulary tests (described below).

Main exclusion criteria were (a) no control group population, (b) the presence of any pathological status in the offspring that might interfere to the resulting cognitive ability scores in infant/child, and (c) pre-clinical studies.

### Data extraction and statistical analysis

Authors M.C.R. and M.J.M. extracted all cognitive-related data from eligible studies. A third author (L.G.F.) checked the data extraction and entered them into Review Manager (RevMan 5.1) and GRADEpro 3.6. Data from RevMan were used to perform the meta-analysis by the Meta and Meta for R packages.

The cognitive effect was assessed using the standardized mean differences (SMD) and their 95% confidence interval (CI) in order to work with the effect sizes and to consider the possible variations. For an effect-size of 0.3 with an average n per group of study 40 and low degree of heterogeneity, 7 studies are needed for obtaining a power of 80%. Data by outcome were statistically combined if they were available and of sufficient quality. Combined means were obtained in a random effect model by using the DerSimonian-Laird method [24]. Random effect models to compute the summary SDM were applied. The random effect model is considered as appropriate since it was not reasonable to assume that studies were estimating the same underlying effect mainly due to possible differences in group population and maternal diabetes type. Meta-regression was performed with those covariates to quantify the hypothesized heterogeneities and to take into account potential confounders [25,26]. Subgroup analysis of meta-regression was performed for those outcomes measured by different types of questionnaire/scales.

We calculated the Q and I^2^ statistics to examine heterogeneity across studies. I^2^ can be interpreted as the proportion of total variation explained by between-study variation [27]. The normality assumption of outcomes was checked and residual of the meta-analysis adjusted and unadjusted were assessed visually for asymmetry (interpretable as publication bias) by using funnel plots and for normality by using Q-Q plots. Potential publication bias was assessed by visual inspection of Begg´s funnel plots and by the Begg’s rank correlation and Egger’s linear regression tests [28,29].

Sensitivity analysis was carried out using cumulative meta-analysis in order to examine the influence of a single study on the combined SMD, by omitting one study and analyzing the remaining ones each turn.

All statistical tests were two-sided and p<0.05 was considered statistically significant except for the heterogeneity Q test in which a p = 0.1 was used in case of a small number of trials [30,31].

### Quality assessment

Three authors (M.C.R., L.G.F. and M.J.M.) evaluated the quality appraisal and graded the risk of bias of the included studies, independently. Quality assessment of the methodology was performed using the Newcastle-Ottawa scale (NOS scale) [32] and risk of bias was assessed by GRADE approach [33]. Questions about selection, assessment of exposure and of prognostic factors, adjustment for all variables, assessment of outcomes and follow-up were evaluated for each study. Studies were classified as at low, moderate, or high risk of bias according to the criteria defined by GRADE.

## Results

**Fig 1:**
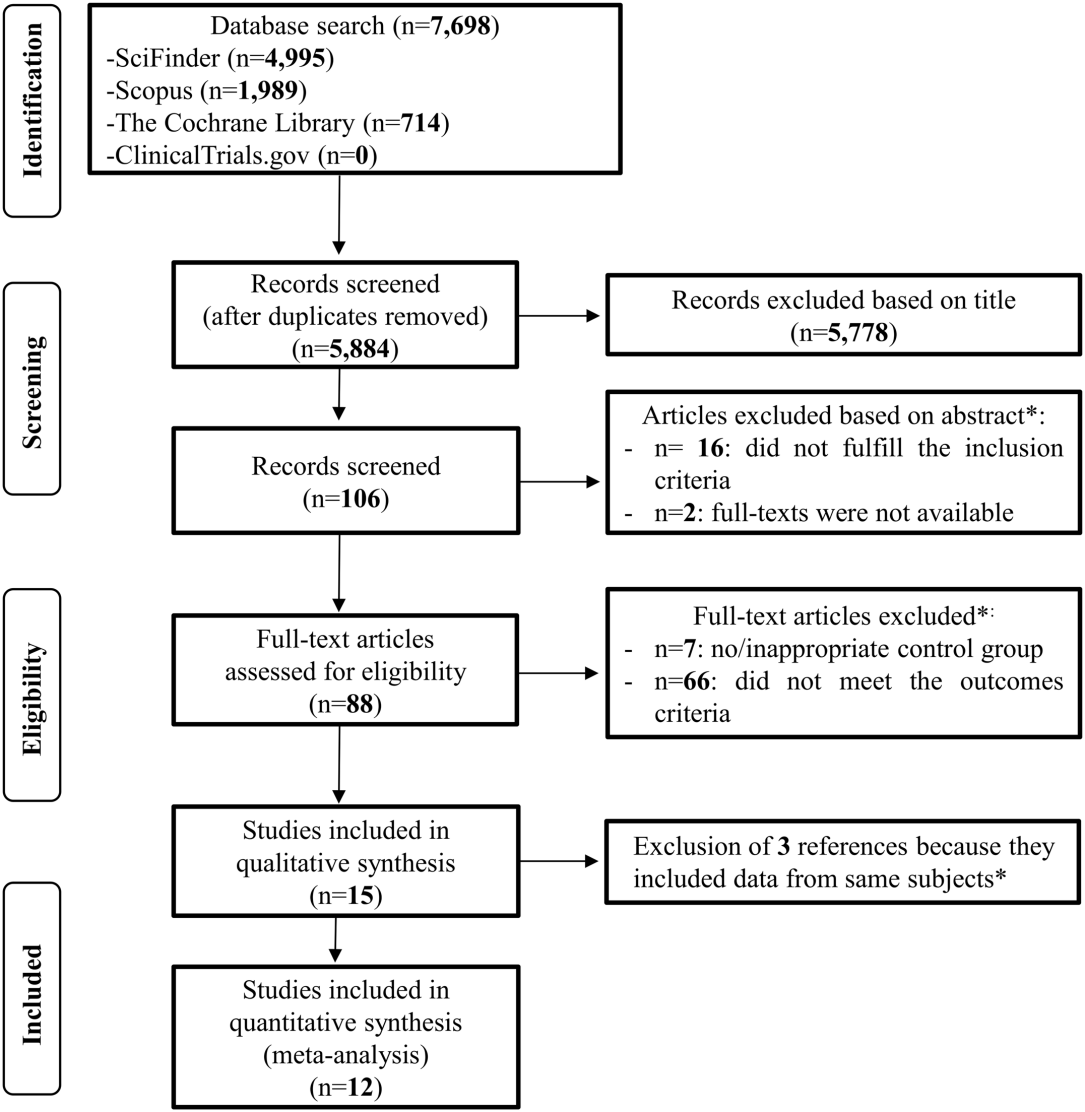
Study flowchart showing the number of studies identified, screened, assessed for eligibility and included in the qualitative and quantitative analysis [17]. *Further information regarding the excluded studies can be found in S2 Text.

Fig 1 shows the flowchart of the systematic literature review and meta-analysis. A total number of 7,698 records were identified through database searches. After initial screening, 88 full-text articles were retrieved. Of these, 73 studies were excluded as they did not meet the inclusion criteria (S2 Text).

A total of 15 studies were included in the systematic review and used for subsequent qualitative and quantitative assessment. No randomized controlled trial (RCT) assessing the long-term effects of pregnancy diabetes on offspring cognition was found during our extensive literature search. Upon further examination, 3 studies were found to include the same subjects [34,35,36] thus, only data from the oldest study were extracted and included in the meta-analysis [34]. In addition, we identified 2 studies providing data apparently from the same cohort population, but contacted corresponding author recommended treating the information as cross-sectional [14,37]. The total number of datasets included in the meta-analysis was therefore 12. A summary of extracted data of included studies is given in Table 1.

**Table 1:** Characteristics of the cohort studies included in the systematic review and meta-analysis (*n* = 12).

Reference and type of study	Maternal age	Maternal BMI	Maternal diabetes diagnosis	Diabetes control	Sample size^†^(*n*)	Child age(years)	SES	Parental education	Other confounders	Cognitive test and primary cognitive outcome*	Findings	Risk of bias	Quality score
Fraser et al. 2012 Cohort prospective, ALSPAC (UK)	ND	GDM women were overweight	Questionnaire	ND	PGDM *n* = 20, GDM *n* = 23, ctrl *n* = 5079	8	Yes	Yes	Sex and maternal age at birth, pre-pregnancy BMI, maternal smoking, parity, mode of delivery, gestational age, birth weight standardized for gestational age and duration of breastfeeding	WISC-III Total IQ: p = 0.09, Ctrl: 105.3 (16.3), PGDM: 103.2 (17.7), GDM: 98.7 (19.9). Adjusted total IQ: Mean differences (95% CI): Ctrl: 0, PGDM: -0.54 (-9.61, 8.52), GDM: -5.93 (-14.24, 2.38)	Support: GDM and PGDM were consistently associated with lower offspring cognition	L	8
Nomura et al. 2012 Cohort retrospective (Queens College, Flushing, New York, USA)	ND	ND	Mother’s retrospective report	ND	GDM *n* = 12, ctrl *n* = 97	4	No	No	Age of mother, mother’s alcohol use and smoking during pregnancy, age, sex, race/ethnicity, and birth weight of the child, and maternal ADHD symptoms, paternal ADHD symptoms, and risk-group status.	WPPSI-III (full-scale) Ctrl: 113.6 (3.5), GDM: 109.2 (1.4), P˂0.001	Support: Children of GDM mothers especially those raised in lower SES showed signs of suboptimal neurocognitive and behavioral development	L	7
Hod et al. 1999 Cohort prospective (Israel)	ND	ND	Management and control of maternal diabetes at the Rabin Medical Center	Diet+ insulin	T1DM *n* = 21, T2DM *n* = 10, ctrl *n* = 41	1	No	No	No significant differences in maternal age, gestational age at delivery, incidence of premature delivery, birth weight, or neonatal complications	BSID-II MDI: p˂0.05, Ctrl: 98.15 (12.05), PGDM: 91.04 (9.01). PDI: p˂0.05, Ctrl: 95.54 (18.14), PGDM: 85.15 (14.53). By subgroup: MDI: N.S., T1DM: 90.4 (8.4), T2DM: 92.1 (10.3). PDI: p˂0.01, T1DM: 89.3 (12.8), T2DM: 78.1 (15.2)	Support: Infants of PGDM women scored lower on mental and psychomotor measures	M	5
Yamashita et al. 1996 Cohort prospective (Kurume, Japan)	DM: 30.6 years; Ctrl: 29.6 years	ND	OGTT	Diet *n* = 7 Diet+insulin *n* = 26	T2DM *n* = 24, T1DM *n* = 6, GDM *n* = 3, ctrl *n* = 34	3–4	No	No	No significant differences in birth weight, in duration of pregnancy, maternal age and age at time of IQ testing, but there were in duration of pregnancy and maternal toxoanemia	Tanaka-Binet Intelligence scale Ctrl: 113.4 (15.3), DM: 98.4 (17.4), p˂0.0001	Support: The offspring of DM mothers showed a poorer intellectual development than ctrls	H	6
DeBoer et al. 2005 Cohort prospective (Minnesota, USA)	ND	ND	OGTT	Diet with or without insulin	DM *n* = 13, ctrl *n* = 16	Up to 1	No	No	Gestational age	BSID-II MDI: p˂0.05, Ctrl: 103 (10), DM: 95 (8); F (1,27) = 5.50. PDI: p = 0.06, Ctrl: 102 (13), DM: 89 (21); F (1,26) = 3.93	Mixed: Significant differences were found on the MDI scale, but not on the PDI	H	7
Nelson et al. 2003 Cohort prospective (Minnesota, USA).	ND	ND	OGTT	Diet with or without insulin	DM *n* = 52, ctrl *n* = 75.	1	No	Yes	No significant differences in gestational age, and maternal age, but there were in birth weight	BSID-II MDI: p˂0.03, Ctrl: 104 (8), DM: 100 (9). PDI: N.S., Ctrl: 101 (13), DM: 98 (17)	Mixed: Children of DM mothers showed significantly lower MDI scores, but no differences on the PDI	L	7
Ornoy et al. 1998^§^ Cohort retrospective (Israel)	ND	ND	Laboratory examinations	Low sugar diet and insulin	PGDM *n* = 57, ctrl *n* = 57	5–12 (mean 8)	No	Yes	Children were matched by age and school placement, by gestational age and birth order	WISC-R Total IQ: p = 0.6, Mean (SE):Ctrl: 118.5 (1.3), PGDM: 117.7 (1.7)	Mixed: Pregnancy diabetes adversely affected some fine neurological functions (motor), but not their IQ- cognitive scores. No correlation with the degree of glycemic control	L	7
Sells et al. 1994 Cohort 3-year follow-up (University of Washington) in the collaborative DIEP (USA)	T1DM: 26.6 years Ctrl: 30.5 years	ND	Described in detail elsewhere^35^	Better in “early entry” mothers. “Late entry” mothers had significantly higher mean glycosylated hemoglobin during the 1^st^ and 2^nd^ trimesters than “early entry”	For MDI: T1DM *n* = 93, ctrl *n* = 83. For PDI: T1DM *n* = 93, ctrl *n* = 83. For IQ: T1DM *n* = 62, ctrl *n* = 65	1, 2 and 3	No	Yes	Not adjusted	BSID-I MDI: *1 year*: N.S., Ctrl: 117 (12.5); T1DM “early entry”: 113 (15.3), T1DM “late entry”: 112 (13.5). *2 years*: N.S., Ctrl: 118 (18.4), T1DM “early entry”: 118 (19.4), T1DM “late entry”: 112 (16.8). PDI: *1 year*: N.S., Ctrl: 103 (15.8), T1DM “early entry”: 104 (15.5), T1DM “late entry”: 102 (17.2). *2 years*: N.S., Ctrl: 110 (18.2), T1DM “early entry”: 108 (17.5), T1DM “late entry”: 112 (21.5). Stanford-Binet Intelligence Scale (4th Edition) IQ:*3 years*: N.S., Ctrl:110 (9.6), T1DM “early entry”: 109 (7.9), T1DM “late entry”: 103 (11)	Mixed: T1DM mothers who maintained good glycemic control during pregnancy can expect to have infants with a normal neurodevelopment as compared to those whose diabetes is less well controlled Non-significant differences were found	L	9
Rizzo et al. 1991 Cohort prospective (Prentice Women's Hospital, Chicago, IL, USA)	ND	ND	OGTT	The “high risk” group received insulin	For MDI: PGDM *n* = 75, GDM *n* = 82, ctrl *n* = 29. For IQ: PGDM *n* = 80, GDM *n* = 79, ctrl *n* = 27	3–5	Yes	No	Race or ethnicity origin	BSID-I MDI: *2 years*: N.S., Ctrl: 89 (13), PGDM: 89 (18), GDM: 90 (14). Stanford-Binet Intelligence Scale IQ: *Average score at 3*,*4 and 5 years*: N.S, Ctrl: 92 (10), PGDM: 89 (14), GDM: 93 (11)	Mixed: Inverse correlations between MDI scores and plasma β-hydroxybutyrate. Inverse correlations of Stanford-Binet scores with β-hydroxybutyrate and free FA levels of the mother. No differences in cognitive function.	L	8
Townsend et al. 2005 Cohort retrospective (Minnesota, USA)	ND	ND	ND	ND	DM *n* = 15, ctrl *n* = 15	4	No	No	Gestational age	WPPSI-R IQ: N.S, Ctrl:121 (21), DM:118 (15).	No support: “Low-risk” children born to DM mothers (good glycemic control) demonstrated typical long-term neurocognitive development.	M	5
Nelson et al.2000 Cohort prospective (University of Minnesota, USA)	ND	ND	OGTT	Diet with or without insulin	DM *n* = 32, ctrl *n* = 25	1	No	No	Not adjusted	BSID-II MDI: N.S., Ctrl: 105 (8.7), DM: 103 (7.5). PDI: N.S., Ctrl:102 (11.9), DM: 102 (9.5)	No support: The BSID exam failed to distinguish the effect between both children groups.	H	6
De Regnier et al. 2000 Cohort prospective (New York, USA)	ND	ND	Medical history	Only *n* = 12 treated with insulin	DM *n* = 22, ctrl *n* = 27	1	No	Yes	No significant differences in gestational age, birth weight and birth head circumference	BSID-II MDI: p = 0.31, Ctrl: 104.7 (1.6), DM: 102.6 (1.4). PDI: p = 0.31, Ctrl: 103.4 (1.9), DM: 100.6 (3.4)	No support: No difference in MDI or PDI scores nor in the language examination	H	4

Studies list is ordered by date and by type of outcome (Most recent studies and “support” are showed first).

ALSPAC, Avon Longitudinal Study of Parents and Children; ND, Not defined; GDM, Gestational Diabetes Mellitus; PGDM, Pre-gestational Diabetes Mellitus; Ctrl, Control; BMI, Body Mass Index; WISC-III/R, Wechsler Intelligence Scale for Children-3^rd^ Edition, R, revised; IQ, Intelligence Quotient; ADHD, Attention Deficit Hyperactivity Disorder; WPPSI-III/R, Wechsler Preschool and Primary Scale of Intelligence–3^rd^ Edition, R, Revised; SES, Socio Economic Status; T1DM, Type 1 Diabetes Mellitus; T2DM, Type 2 Diabetes Mellitus; BSID-I/II, Bayley Scales of Infant Development- 1^st^/2^nd^ Edition; MDI, Mental Development Index; PDI, Psychomotor Development Index; OGTT, Oral Glucose Tolerance Test; DIEP, Diabetes in Early Pregnancy; FA, Fatty Acids.

Risk of bias classification (GRADE): L, Low; M, Medium; H, High.

Quality score (Newcastle-Ottawa): from 0 (lowest) to 9 (highest).

^†^ Number of children included in the studies.

*All values refer to mean (Standard Deviation), otherwise it is stated.

^§^ Three references are related to the same subjects, so only the oldest study was included.

Most of the studies were of high quality according to NOS (Table 1 and S1 Table) and of low risk by GRADE questionnaire (S2 Table). Publication bias was assessed using rank correlation test and regression test for funnel plot asymmetry. Neither asymmetry nor significant bias were found (S3 Table and S1 Fig).

Of the 12 studies included in the meta-analysis, 9 were prospective longitudinal cohort studies [12,14,37,38,39,40,41,42,43] and 3 were retrospective cohort studies [11,13,34].

The mental and psychomotor development were examined at age 1–2 years [14,37,38,39,40,42,43], while intellectual ability yielded by IQ measures was monitored at age 3–12 years [11,12,13,34,38,39]. Two studies performed both cognitive measures [38,39]. While 4 studies supported a general negative impact of maternal diabetes on child neurodevelopment [11,12,40,41], 5 reported mixed results showing a negative effect on a particular cognitive domain [34,38,39,42,43] and 3 did not found a consistent adverse effect [13,14,37] (Table 1).

### Mental and psychomotor development outcomes in infancy

The systematic review identified 7 studies reporting measures of MDI (in BSID-I and -II) [14,37,38,39,40,42,43] which were included in the meta-analysis. All of them evaluated the MDI at age 12 months, except Rizzo et al. [38] who reported data at age 24 months. All children from diabetic mothers were considered as one group since no differences between them were found when adjusting for the type of diabetes.

**Fig 2:**
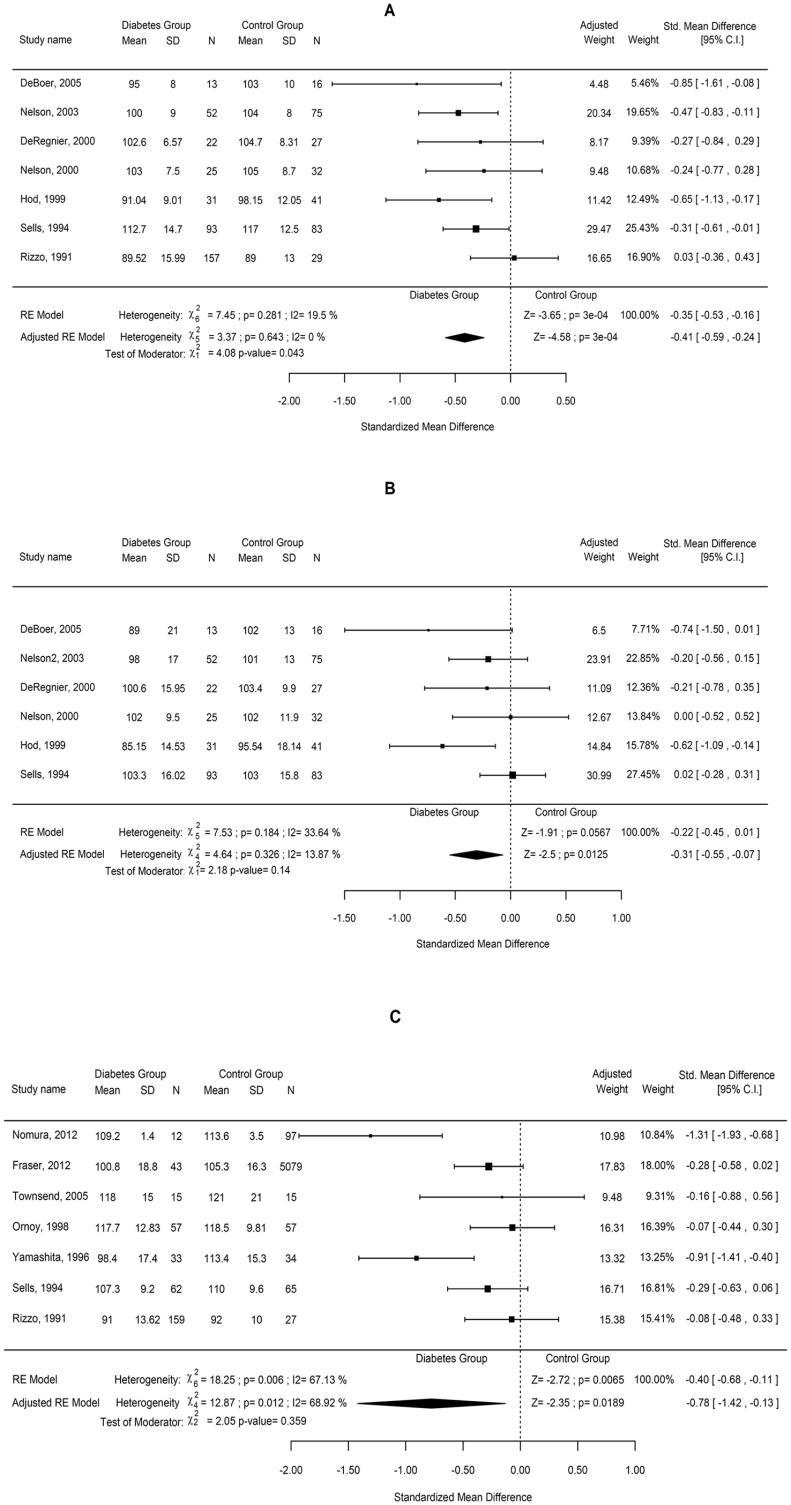
Effect of pregnancy diabetes on the mental and psychomotor development and intelligence quotient. Forest plots comparing the difference in the (A) Mental Development Index and (B) Psychomotor Development Index subscales of the BSID between children of diabetic and non-diabetic mothers. (C) Forest plots comparing the difference in the intelligence quotient yielded by combined data from the Wechsler scales and the Stanford-Binet intelligence scale between children of diabetic and non-diabetic mothers.

Data from the 7 studies were combined using random effect models and the results are shown in Fig 2A. The combined SMD and the 95% CIs are presented using both a univariate and a multivariate random effect model adjusted by the age of infants. Although for the univariate model the χ^2^ test for heterogeneity showed a non-significant result, the I^2^ index showed low to moderate non-significant variation (19.5%). Variation disappeared (I^2^ = 0) after adjusting by age, showing a significant effect of this variable (p = 0.043). The combined effect was 0.41 lower for those infants of diabetic mothers, being the results significant at 5% level and with 95% CI (-0.59, -0.24).

The sensitivity analysis showed no relevant changes on the combined results by study deletions (narrow range of estimated SMD). The estimated effect ranged from -0.47 to -0.38, suggesting that the significant effect is not determined by a single study. Thus, the findings were robust against study deletions. Further analysis including all the covariates is shown in S4 Table.

The systematic review identified 6 studies examining the PDI (in BSID-I and -II) [14,37,39,40,42,43] that were used to meta-analysis. We found that children of diabetic mothers had lower PDI than those in control group, with an average standardized difference of 0.31 and 95% CI (-0.55, -0.07) (Fig 2B). The association between pregnancy diabetes and decreased PDI remained significant after adjusting for type of maternal diabetes in those studies where it was differentiated and diabetes control when stated (p values of 0.429 and 0.961, respectively). Moreover, heterogeneity among studies was not changed by adjustment for these confounding factors. Cumulative meta-analysis over time showed significant effects when adding the most recent studies (S5 Table). Thus, the year of the study was also considered as a covariate for meta-regression, but not significant association was found (p = 0.170).

### Intelligence quotient outcome in childhood

Seven studies reported data regarding IQ and were included in the meta-analysis; 4 studies reported IQ scores measured by Wechsler scales [11,12,13,34] and 3 reported IQ based on Stanford-Binet test [38,39,41].

A general result was initially obtained from all studies, adjusting by type of questionnaire and child age. Since children were examined at different ages, two age groups were considered for calculation, age 3–5 years and age 5–12 years. However, results from meta-regression showed no significant effect of the moderators considered and no improvement of the unexplained variation on the results (I^2^ = 68.92%) (Fig 2C). A significant IQ reduction (SMD = -0.78; CI -1.42, -0.13; p = 0.0189) in diabetes-exposed children was found, suggesting a potential adverse effect of maternal diabetes on child intellectual function. Adjustment by type of study design, type of maternal diabetes or presence/absence of diabetic control was considered in the meta-regression model. However, no significant association among those factors was found (p values of 0.339, 0.707 and 0.473, respectively) and no reduction in the heterogeneity was observed. Further cumulative meta-analysis is showed in S6 Table.

In attempting to reduce variability, two subgroups were meta-analyzed for the IQ outcome taking into account the type of test: one for the Wechsler scales and another for the Stanford-Binet test (S2A and S2B Fig). Significant variation was also found in the subgroup analyses. The age-adjusted results for the subgroup of studies using the WISC test showed significantly lower IQ values for the children in the diabetes group (-0.78; CI -1.42, -0.13, p = 0.0187) but with medium significant variation (I^2^ = 68.3%) (S2A Fig). In contrast, the combined result for the subgroup of studies using the Stanford-Binet scale showed no significant effect (S2B Fig). That indicated that neither the type of questionnaire nor the children age was contributing to the significant heterogeneity observed.

The sensitivity analysis showed relevant changes on the combined results by study removals, ranging the estimates effects from -1.13 to -0.16 for the adjusted model, and from -0.46 to -0.28 for the unadjusted results. Thus, the findings were not robust against study deletions. Conversely to control, IQ values in the children of diabetic mothers group were highly variable, ranging from average to lower than average scores, which may account for the increased heterogeneity.

### Correlation between maternal diabetes type and children’s cognition

Although, the majority of the studies did not differentiate between type of diabetes, Hod et al. [40] found that T2DM-exposed infants scored lower on the PDI than those exposed to T1DM, but higher on the motor quality score as compared to control children. When T1DM- and T2DM-exposed children were grouped together in the study of Ornoy et al. [34], it was showed that sensory-motor function of index children tended to be lower with higher glycosylated hemoglobin. Rizzo et al. [38] compared the effect of PGDM (without diabetes type discrimination) and GDM exposure in children of age 1 year and 3–5 years. They found that both diabetic exposed-children showed similar defective behavioral and intellectual development, although iron deficiency and neonatal hyperglycemia were prevalent in infants of preexisting diabetes mothers. A group of longitudinal studies explored the effect of maternal glycemia status on early age children (1 year) [13,14,37,43]. They found significant lower cognitive scores in diabetes-exposed children (without differentiating preexisting or gestational) based on behavioral BSID measures which correlated with electrophysiological recordings suggesting that the observed adverse effects might be mediated by damage of the hippocampus.

Two studies suggested that children of mothers with well-managed diabetes during pregnancy showed better cognitive performance (in MDI, PDI or IQ) than those of mothers with a suboptimal glucose management [13,39]. Sells et al. [39] in a 3-year follow up study suggested that children of mothers recruited at early pregnancy and presenting a well-managed T1DM showed better MDI, PDI, and IQ scores than those recruited later, who did not keep optimal blood glucose levels for most of the pregnancy. Conversely, no correlation between IQ scores and blood glucose and maternal hemoglobin A_1c_ levels during pregnancy was found by Ornoy et al. [34] and Yamashita et al. [41].

The type of maternal diabetes was considered as a potential factor influencing the cognitive outcomes of the offspring. However, when data from children born to T1DM, T2DM, and GDM mothers were compared, no significant differences were found with respect to the analyzed outcomes. Therefore, all diabetic mothers were considered as an only group and analyzed together.

### Cognitive outcomes in excluded studies

The systematic review also identified several studies that assessed language performance and other relevant cognitive abilities in children of pregnant women with and without diabetes, but insufficient number of studies did not allow us to use meta-analysis.

For instance, poorer language development was observed in children of diabetic mothers scored by NEPSY (Neuropsychological) evaluation [11], Peabody Picture Vocabulary (PPV) test [39] and McArthur Communicative Development Inventory (MCDI) test [37,44]. Three studies identified in the systematic review [11,39,44] found that children of diabetic mothers showed language deficiencies, while only that by DeRegnier et al. [37] did not find any difference as compared to control.

Some differences regarding motor development were found in children of preexisting diabetes by using the Bruininks-Oseretsky of motor proficiency test, but this test was exclusively used by this group and comparison to the others tests was not possible [34]. Electrophysiological recordings of even-related potentials (ERP) in diabetes-exposed infants demonstrated strong deficits in recognition (explicit) memory [14,37,42,43]. More severe effects of maternal diabetic condition have been observed in children who presented increased risk of neurodevelopmental disorders including ADHD [11,34] and autism [45].

## Discussion

This systematic review and meta-analysis found that infants of diabetic mothers have a lower mental and psychomotor development than non-diabetes exposed infants at age 1–2 years. There was some evidence of a low IQ score observed in children (3–12 years) of women with pregnancy diabetes, but significant heterogeneity did not allow drawing conclusions. Other cognitive abilities were adversely affected among children of diabetic mothers including language development and motor performance; however, there were insufficient studies to draw conclusions about these outcomes. Deleterious effects of diabetes during pregnancy had already been suggested by other authors [7,16] although to our knowledge no statistical analysis has been performed to date.

Environmental influence on the child mental development increases as the child grows. Influence of school attendance and teaching quality are closely related to socioeconomic status (SES). Among all socioeconomic factors, maternal education level is the one that affects the most to child cognitive development [46]. Thus, among children of diabetic mothers, those from a low SES scored lower in all cognitive outcomes including IQ when compared to high SES children [11]. In fact, in a study performed in two Indian cohorts, children from diabetic mothers performed better in the different cognitive tests than those from control mothers, likely due to a higher maternal education, urban residence and a better nutritional status of the former children [15]. However, when diabetic and control mothers came from a similar cohort, sharing the same SES, the differencies between their offspring cognitive abilities were not significant [47]. Six studies in this meta-analysis did not adjust by SES and parental education [11,13,14,40,41,43]. The rest of included studies adjusted data for SES or parents’ educational level, thus reported findings must be considered as independent of this confounder (see Table 1). Moreover, these variables were taken into account when recruiting the mothers, but no information was provided about the education of the child.

Some published studies compared school marks of children exposed or not to diabetes during gestation, although we did not consider them for meta-analysis due to lack of standardization among scores. Thus, Fraser et al. [12] evaluated the effect of early life exposure to high glucose levels in the mother and child's IQ scores (at 8 years) in the Avon Longitudinal Study of Parent and Children (ALSPAC) cohort and showed that maternal impaired glycemic status (PGDM or GDM) was consistently associated with worse offspring school entry assessment scores such as IQ and General Certificate of Secondary Education (GCSE) scores. Conversely, the evaluation of the cognitive performance in the adult offspring of diabetic mothers failed to find an association between GDM and cognitive test scores [48,49]. According to this and our results, it could be hypothesized that the influence of diabetes during gestation on offspring cognition could be important in the youngest ages and then progressively would diminish as the individual grows.

With respect to the type of diabetes, most of the studies did not differentiate and consider all diabetic mothers as a unique group. When separated, it was found that offspring from T1DM mothers had a better psychomotor development (PDI) than those from T2DM mothers, although their mental development was similar [40]. Whether this fact is due to a better glycemic control by T1DM women or to differences in the etiology of the disease remains unclear.

Children of mothers presenting a well-managed diabetes during pregnancy showed better cognitive (MDI and PDI) and intellectual (IQ) abilities than those born to women who did not keep optimal blood glucose levels [39]. Moreover, children of T1DM mothers achieving a poor glycemic control either pregestationally or during pregnancy, showed lower school marks than the matched control offspring, and this difference was significant even after adjusting for parental education [47]. Therefore, the potential negative effect of diabetes could be even stronger in populations with a looser control of the glycemia during pregnancy.

### Strengths and limitations

Some recent literature reviews have addressed the influence of maternal diabetes on cognitive development of children [8,16]. The topic is therefore of relevance from the public health perspective and the present meta-analysis can contribute to clarify some of such effects by providing statistical assessment. In addition, included studies were consistent across those outcomes measured in the youngest children since no statistically significant heterogeneity was detected. However, in older children published data were too heterogeneous to reach any conclusion.

The main limitation of this meta-analysis is the lack of existing RCTs comparing the effects of different treatments for controlling glycemia during pregnancy on cognitive development of the offspring [50]. The included epidemiological studies show that children of diabetic mothers have some delay in their cognitive development at early ages. Therefore, the identification of the most efficient treatment could contribute to prevent such undesirable effect.

Another important limiting factor is the unbalanced sample sizes between diabetic and control groups in some of the studies, which may not accurately reflect the effect of the findings in a larger population. Low number of studies could be a further limitation but in our case the effects did not vary substantially among studies.

Although due to methodological limitations most studies did not include information related to mother’s BMI, and lean mothers do also develop GDM, it is well-known that high maternal weight is significantly associated with a higher risk of GDM development [51]. Thus, 2 studies stated the inclusion of overweight diabetic mothers into the studied population [12,15], but only 1 presented adjusted extractable data reporting that overweight and GDM were consistently associated with worse school marks and IQ scores [12]. That is correlated with recent findings of a systematic review which suggested that the offspring of obese pregnancies may be at increased risk of cognitive problems in childhood that may persist till adulthood [52].

In fact, a recent publication from the PREOBE team revealed significant impaired neurodevelopment with regard to language, motor and cognitive scores in infants (age 18 months) of overweight and obese mothers[53]. Infants of GDM mothers also showed significant delays in gross motor development, expressive language and composite language scores but exhibited normal cognitive scores. However, the raw differences in BSID-III scores between 6 and 18 months showed a loss of 1.5 points in the GDM group while the group of non diabetic/normal weight mothers showed an increase of 3 points. This study was not included in our meta-analysis since a different BSID version (third edition) was used which released cognitive scores not comparable to previous versions [54]. So, we cannot rule out the possibility that maternal overweight/obesity may have a synergistic effect in inducing adverse neurodevelopment in children.

While the majority of studies to date were conducted in U.S., we also included studies performed in UK, Japan and Israel. Thus, given the rising problem of preexisting diabetes and GDM globally, more data from different geographical locations may be needed to achieve a worldwide conclusion.

Since this meta-analysis has systematically identified all these limitations, the design of future studies can be substantially improved.

## Conclusion

The prevalence of pregnancy diabetes worldwide continues to be one of the major health concerns for public health. This meta-analysis has identified statistically significant cognitive impairment in infants born to diabetic mothers during their first year, which could mean certain delay in mental performance during school-age. However, results need to be taken with caution since they are based on observational studies and therefore, a cause-effect relationship cannot be established.

Influence of maternal diabetes has not been clearly determined and many other intrinsic and extrinsic factors, i.e. metabolic health status or socio-economic level also contribute to cognitive performance of the child. Therefore, controlled clinical trials would shed light on the question and help designing a better strategy for coping with diabetes during gestation.

## Supporting Information

S1 Fig (A) MDI: Galbraith plot and funnel plot for the unadjusted and adjusted model. (B) IQ: Galbraith plot and funnel plot for the unadjusted and adjusted model. (C) PDI: Galbraith plot and funnel plot for the unadjusted and adjusted model.
(PDF)

S2 Fig Subgroup meta-analysis for the IQ measures yielded by (A) Wechsler scales and (B) Stanford-Binet scale. (PDF)

S1 Table Quality assessment according to NOS scale [32]. (PDF)

S2 Table Risk of bias in included studies according to GRADE approach [33]. (PDF)

S3 Table Rank correlation test and regression test for funnel plot asymmetry. (PDF)

S4 Table Cumulative analysis for the MDI measures. These studies included had in common the age of the infants and so, by including the covariate age in the model, the variability due to the factor was taken into account.
(PDF)

S5 Table Cumulative analysis of the combined data relative to motor function (PDI). (PDF)

S6 Table Cumulative meta-analysis for the combined IQ measures. Cumulative meta-analysis over time shows significant effects when adding later studies. So a covariate with the year of the study was also considered for meta-regression. No significant association of the year (*p*-value 0.329), neither a reduction of the variation was found after adjustment.
(PDF)

S1 Text Databases search strategy. (PDF)

S2 Text Details of excluded studies at the screening and eligibility review stage. (PDF)
