# Supplementary material for: Maternal Diabetes and Cognitive Performance in the Offspring: A Systematic Review and Meta-Analysis
Source: PLoS One. 2015 Nov 13;10(11):e0142583. doi: 10.1371/journal.pone.0142583 (PMC4643884; doi:10.1371/journal.pone.0142583)

S1 Fig. (A) MDI: Galbraith plot and funnel plot for the unadjusted and adjusted model. (B) IQ: Galbraith plot and funnel plot for the unadjusted and adjusted model. (C) PDI: Galbraith plot and funnel plot for the unadjusted and adjusted model.

A

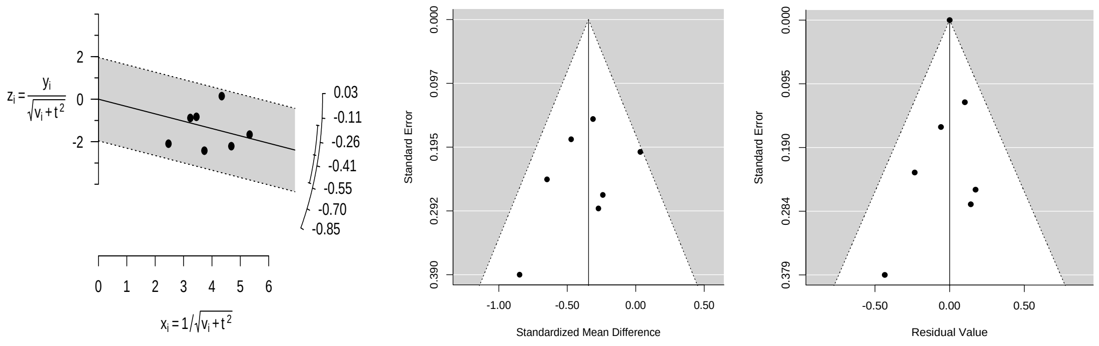

B

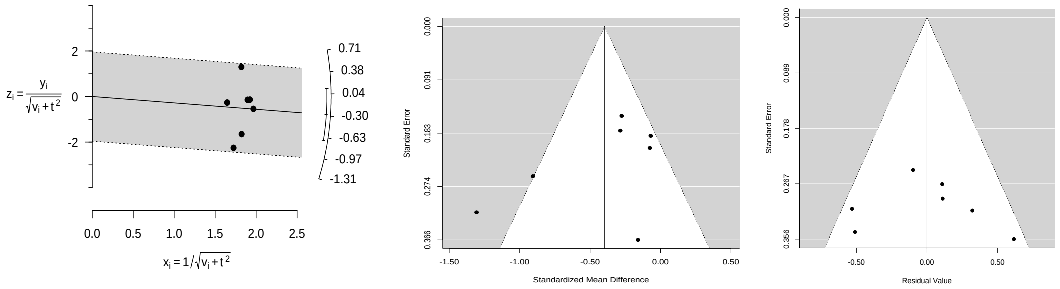

C

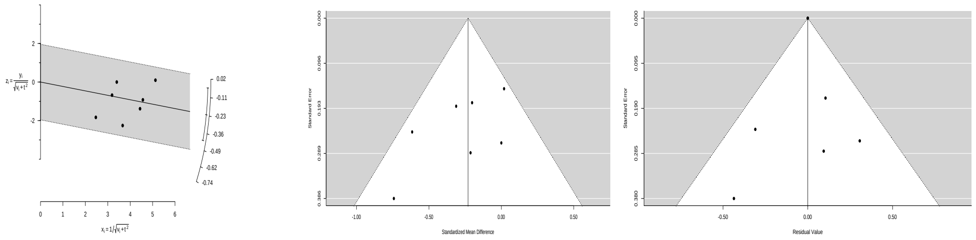

Supplement: S1 Fig — (A) MDI: Galbraith plot and funnel plot for the unadjusted and adjusted model. (B) IQ: Galbraith plot and funnel plot for the unadjusted and adjusted model. (C) PDI: Galbraith plot and funnel plot for the unadjusted and adjusted model. (PDF) [file pone.0142583.s001.pdf]
