# Supplementary material for: Maternal Diabetes and Cognitive Performance in the Offspring: A Systematic Review and Meta-Analysis
Source: PLoS One. 2015 Nov 13;10(11):e0142583. doi: 10.1371/journal.pone.0142583 (PMC4643884; doi:10.1371/journal.pone.0142583)

S2 Fig. Subgroup meta-analysis for the IQ measures yielded by (A) Wechsler scales and (B) Stanford-Binet scale.

A

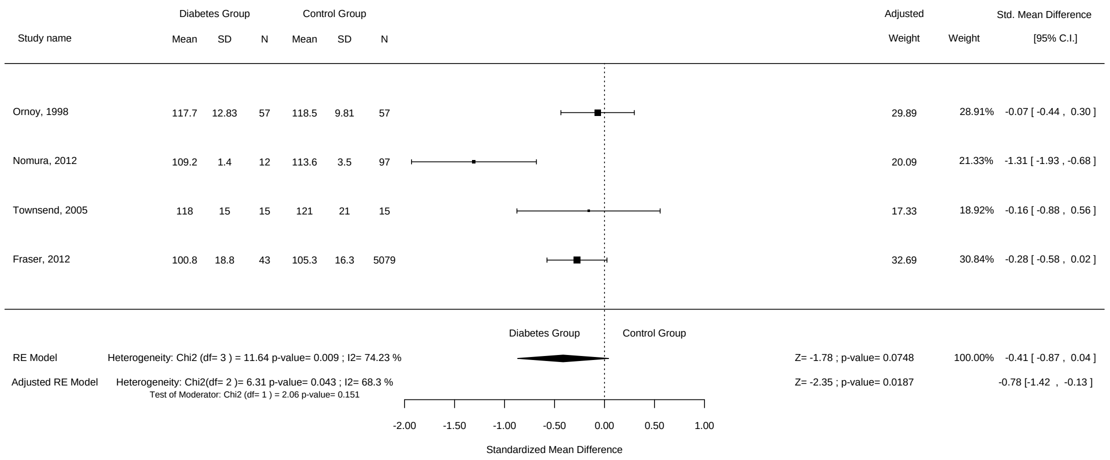

B

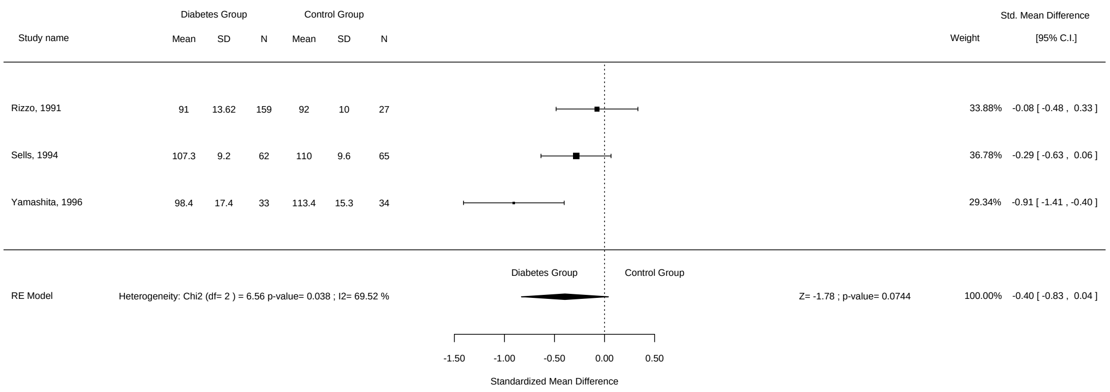

Supplement: S2 Fig — (PDF) [file pone.0142583.s002.pdf]
