# Supplementary material for: Maternal Diabetes and Cognitive Performance in the Offspring: A Systematic Review and Meta-Analysis
Source: PLoS One. 2015 Nov 13;10(11):e0142583. doi: 10.1371/journal.pone.0142583 (PMC4643884; doi:10.1371/journal.pone.0142583)
Supplement: S1 Table — (PDF) [file pone.0142583.s003.pdf]

S1 Table. Quality assessment according to NOS scale. (http://www.medicine.mcgill.ca/rtamblyn/Readings/)

	Selection				Comparability	Outcome			
**Reference**	**Representa tiveness**	**Non exposed cohort**	**Exposure ascertainment**	**Absence of outcome at start**		**Assessment**	**Length of follow-up**	**Adequacy of follow-up**	**Final quality score**
Fraser 2012	*	*	*	*	*	*	*	*	8
Nomura 2012	*	*	*		**	*	*		7
Hod 1999	*	*	*		*	*			5
Yamashita 1996		*	*	*	*	*	*		6
DeBoer 2005		*	*	*	*	*	*	*	7
Nelson 2003	*	*	*	*	*	*	*		7
Ornoy1998	*	*	*	*	*	*	*		7
Sells 1994	*	*	*	*	**	*	*	*	9
Rizzo 1991	*	*	*	*	*	*	*	*	8
Townsend 2005		*	*	*	*		*		5
Nelson 2000		*	*	*	*	*	*		6
De Regnier 2000		*		*	*		*		4
