# Supplementary material for: Maternal Diabetes and Cognitive Performance in the Offspring: A Systematic Review and Meta-Analysis
Source: PLoS One. 2015 Nov 13;10(11):e0142583. doi: 10.1371/journal.pone.0142583 (PMC4643884; doi:10.1371/journal.pone.0142583)
Supplement: S2 Table — (PDF) [file pone.0142583.s004.pdf]

S2 Table. Risk of bias in included studies according to GRADE approach*.

Study	Selection	Assessment of exposure	Outcome not present at start	Adjustment for all variables	Assessment of prognostic factors	Assessment of outcome	Follow up	Overall risk of bias
Fraser 2012	Low	Low	Low	Low	Low	Low	Low	Low
Nomura 2012	Low	Low	Low	Low	Low	Low	NA	Low
Hod 1999	Low	Low	Low	Low	Low	High	NA	Moderate
Yamashita 1996	Low	Low	Low	High	High	Low	Moderate	High
DeBoer 2005	Low	Low	Low	High	High	Low	NA	High
Nelson 2003	Low	Low	Low	Low	Moderate	Low	NA	Low
Ornoy 1998	Low	Low	Low	Low	Low	Low	NA	Low
Sells 1994	Low	Low	Low	Low	Low	Low	Low	Low
Rizzo 1991	Low	Low	Low	Low	Low	Low	Low	Low
Townsed 2005	Low	Low	Low	Moderate	Moderate	Low	NA	Moderate
Nelson 2000	Low	Low	Low	High	High	Low	NA	High
DeRegnier 2000	Low	High	Low	High	High	High	NA	High

* Higgins JPT AD, Sterne. Chapter 8: Assessing risk of bias in included studies. In: Higgins JPT, Green S (editors). Cochrane Handbook for Systematic Reviews of Interventions. Version 5.1.0. 2011.
