# Supplementary material for: Maternal Diabetes and Cognitive Performance in the Offspring: A Systematic Review and Meta-Analysis
Source: PLoS One. 2015 Nov 13;10(11):e0142583. doi: 10.1371/journal.pone.0142583 (PMC4643884; doi:10.1371/journal.pone.0142583)
Supplement: S3 Table — (PDF) [file pone.0142583.s005.pdf]

S3 Table. Rank correlation test and regression test for funnel plot asymmetry.

	Funnel plot Asymmetry							
	Unadjusted				Adjusted			
	Regression test		Rank correlation test		Regression test		Rank correlation test	
**Cognitive outcome**	**Z**	**p-value**	**Kendall´s tau**	**p-value**	**Z**	**p-value**	**Kendall´s tau**	**p-value**
**MDI**	-0.911	0.362	-0.143	0.773	-0.808	0.419	-0.143	0.773
**PDI**	-1.669	0.095	-0.429	0.239	-0.793	0.428	-0.429	0.239
**IQ**	-1.510	0.131	-0.238	0.562	-0.075	0.940	-0.238	0.562
