# Supplementary material for: Maternal Diabetes and Cognitive Performance in the Offspring: A Systematic Review and Meta-Analysis
Source: PLoS One. 2015 Nov 13;10(11):e0142583. doi: 10.1371/journal.pone.0142583 (PMC4643884; doi:10.1371/journal.pone.0142583)
Supplement: S4 Table — These studies included had in common the age of the infants and so, by including the covariate age in the model, the variability due to the factor was taken into account. (PDF) [file pone.0142583.s006.pdf]

S4 Table. **Cumulative analysis for the MDI measures.** These studies included had in common the age of the infants and so, by including the covariate age in the model, the variability due to the factor was taken into account.

Study	Estimate	S.E.	p-values	95% C.I. Lower Limit	95% C.I. Upper Limit
Rizzo 1991	0.0333	0.2021	0.8693	-0.3629	0.4294
Sells 1994	-0.1653	0.1709	0.3335	-0.5002	0.1696
Hod 1999	-0.2909	0.1743	0.0952	-0.6325	0.0508
DeRegnier 2000	-0.2837	0.1333	0.0333	-0.5449	-0.0224
Nelson 2000	-0.2747	0.1059	0.0095	-0.4823	-0.0670
Nelson 2003	-0.3159	0.0911	0.0005	-0.4944	-0.1373
DeBoer 2005	-0.3452	0.0946	0.0003	-0.5307	-0.1596
