# Supplementary material for: Maternal Diabetes and Cognitive Performance in the Offspring: A Systematic Review and Meta-Analysis
Source: PLoS One. 2015 Nov 13;10(11):e0142583. doi: 10.1371/journal.pone.0142583 (PMC4643884; doi:10.1371/journal.pone.0142583)
Supplement: S5 Table — (PDF) [file pone.0142583.s007.pdf]

S5 Table. Cumulative analysis of the combined data relative to motor function (PDI).

Study	Estimate	S.E.	p-values	95% C.I. Lower Limit	95% C.I. Upper Limit
Sells 1994	0.0188	0.1510	0.9011	-0.2772	0.3147
Hod 1999	-0.2698	0.3160	0.3932	-0.8892	0.3496
DeRegnier 2000	-0.2384	0.2021	0.2380	-0.6345	0.1576
Nelson 2000	-0.1768	0.1518	0.2440	-0.4743	0.1207
Nelson 2003	-0.1720	0.1104	0.1192	-0.3884	0.0444
DeBoer 2005	-0.2217	0.1163	0.0567	-0.4497	0.0063
