# Supplementary material for: Maternal Diabetes and Cognitive Performance in the Offspring: A Systematic Review and Meta-Analysis
Source: PLoS One. 2015 Nov 13;10(11):e0142583. doi: 10.1371/journal.pone.0142583 (PMC4643884; doi:10.1371/journal.pone.0142583)
Supplement: S6 Table — Cumulative meta-analysis over time shows significant effects when adding later studies. So a covariate with the year of the study was also considered for meta-regression. No significant association of the year (p-value 0.329), neither a reduction of the variation was found after adjustment. (PDF) [file pone.0142583.s008.pdf]

S6 Table. Cumulative meta-analysis for the combined IQ measures. Cumulative meta-analysis over time shows significant effects when adding later studies. So a covariate with the year of the study was also considered for meta-regression. No significant association of the year (*p*-value 0.329), neither a reduction of the variation was found after adjustment.

Study	Estimate	S.E.	p-values	95% C.I. Lower Limit	95% C.I. Upper Limit
Rizzo 1998	-0.0756	0.2082	0.7164	-0.4837	0.3324
Sells 1994	-0.1965	0.1355	0.1469	-0.4620	0.0690
Yamashita 1996	-0.3964	0.2222	0.0744	-0.8318	0.0391
Ornoy 1998	-0.3044	0.1690	0.0717	-0.6357	0.0269
Townsend 2005	-0.2836	0.1451	0.0506	-0.5680	0.0007
Nomura 2012	-0.4344	0.1847	0.0187	-0.7964	-0.0724
Fraser 2012	-0.3971	0.1458	0.0065	-0.6829	-0.1113
