# Supplementary material for: Maternal Diabetes and Cognitive Performance in the Offspring: A Systematic Review and Meta-Analysis
Source: PLoS One. 2015 Nov 13;10(11):e0142583. doi: 10.1371/journal.pone.0142583 (PMC4643884; doi:10.1371/journal.pone.0142583)
Supplement: S1 Text — (PDF) [file pone.0142583.s009.pdf]

S1 Text. Databases search strategy.

Our search strategy included key terms that are summarized as follows:

#1 ((TITLE-ABS-KEY (“gestational diabetes” OR “diabetic mother” OR “maternal type 1 diabetes mellitus” OR “maternal type 2 diabetes mellitus” OR “diabetes pregnancy”)) AND (TITLE-ABS-KEY (infant* OR child* OR baby OR babies OR newborn* OR toddler* OR preschool* OR pre-school*))).

#2 ((TITLE-ABS-KEY (“gestational diabetes” OR “diabetic mother” OR “maternal type 1 diabetes mellitus” OR “maternal type 2 diabetes mellitus” OR “diabetes pregnancy”)) AND (TITLE-ABS-KEY (“offspring psychomotor function” OR “offspring cognit*” OR “children cognit*” OR cognit*)) AND (TITLE-ABS-KEY (performance OR function OR development)) AND (TITLE-ABS-KEY (“child* behavior” OR development)) AND (TITLE-ABS-KEY (school OR learning OR academic OR reading OR maths OR brain))).

#3 (TITLE-ABS-KEY (“insulin resistance” OR “insulin pregnancy” OR “insulin gestation”) AND (TITLE-ABS-KEY (infant* OR child* OR baby OR babies OR newborn* OR toddler* OR preschool* OR pre-school*)) AND (TITLE-ABS-KEY (“offspring cognit*” OR “children cognit*” OR “cognit*)) AND (TITLE-ABS-KEY (performance OR function OR development)) AND (TITLE-ABS-KEY ((“child* behavior” OR development) AND (school OR learning OR academic OR reading OR math OR brain)))).

#1 AND #2 AND #3.
