# Supplementary material for: Maternal Diabetes and Cognitive Performance in the Offspring: A Systematic Review and Meta-Analysis
Source: PLoS One. 2015 Nov 13;10(11):e0142583. doi: 10.1371/journal.pone.0142583 (PMC4643884; doi:10.1371/journal.pone.0142583)
Supplement: S2 Text — (PDF) [file pone.0142583.s010.pdf]

S2 Text. Details of excluded studies at the screening and eligibility review stage.

Based on abstract: Eleven articles were reviews and not related to cognitive evaluation in offspring of diabetic mothers [1-11]. Two studies corresponded to descriptive reports [12, 13] and one was an observations letter without extractable data [14]. One was a case-report of one infant [15]. One study corresponded to a preclinical study [16]. Two full-text articles were not available [17, 18].

Based on full-text read: Twenty-three studies were excluded because did not include a diabetic mothers group [19-41]. Thirty-one studies did not evaluate behavioral cognitive outcomes of our inclusion criteria [42-71]. Three studies corresponded to poster abstracts containing unpublished results [72-74]. Some studies included only a sample of diabetes-exposed children with ADHD[75-77], autism[78], or schizophrenia[79]. Seven studies did not include an appropriate comparison control group [80-86]. No extractable data were found in three studies [87-89]. One study was excluded because included important residual confounding[40]. Three references were discarded because they contained repeated data [90-92].

REFERENCES

1. Georgieff MK. The effect of maternal diabetes during pregnancy on the neurodevelopment of offspring. Minn Med2006 Mar;89(3):44-7.
2. Grayson BE, Kievit P, Smith MS, Grove KL. Critical determinants of hypothalamic appetitive neuropeptide development and expression: Species considerations. Frontiers in Neuroendocrinology2010;31(1):16-31.
3. Sesma HW, Georgieff MK. The effect of adverse intrauterine and newborn environments on cognitive development: the experiences of premature delivery and diabetes during pregnancy. Dev Psychopathol2003 Fall;15(4):991-1015.
4. Fall CH. Fetal malnutrition and long-term outcomes. Nestle Nutr Inst Workshop Ser2013;74:11-25.
5. Ramanathan M, Jaiswal AK, Bhattacharya SK. Hyperglycaemia in pregnancy: effects on the offspring behaviour with special reference to anxiety paradigms. Indian J Exp Biol2000 Mar;38(3):231-6.
6. Ten S, Maclaren N. Insulin resistance syndrome in children. J Clin Endocrinol Metab2004;89(6):2526-39.
7. Liang J, Matheson B, Kaye W, Boutelle K. Neurocognitive correlates of obesity and obesity-related behaviors in children and adolescents. Int J Obes 2014;38(4):494-506.
8. Das U. Perinatal and childhood nutrition and its impact on cognitive function and adult diseases. AgroFOOD industry hi-tech2008;19(4):4-8.
9. Kapustin JF. Postpartum management for gestational diabetes mellitus: policy and practice implications. J Am Acad Nurse Pract2008;20(11):547-54.
10. Ornoy A. Growth and neurodevelopmental outcome of children born to mothers with pregestational and gestational diabetes. Pediatr Endocrinol Rev2005 Dec;3(2):104-13.
11. Rosales FJ, Zeisel SH. Perspectives from the symposium: The role of nutrition in infant and toddler brain and behavioral development. Nutr Neurosci2008;11(3):135-43.

12. Farquhar JW. The child of the diabetic woman. Arch Dis Child1959 Feb;34(173):76-96.
13. Ley S, O'Connor D, Retnakaran R, Hamilton J, Sermer M, Zinman B, et al. Impact of maternal metabolic abnormalities in pregnancy on human milk and subsequent infant metabolic development: methodology and design. BMC Public Health C7 - 5902010;10(1):1-10.
14. Brinciotti M, Matricardi M, Colatrella A, Torcia F, Fallucca F, Napoli A. Effects of maternal diabetes on visual evoked potentials and early psychomotor development of the offspring. Diabetes Care2007 Dec;30(12):e128.
15. Robinson C. Problems in neonatal medicine. 1. The infant of the diabetic mother. J Natl Med Assoc1972;64(3):203-4.
16. Rodekamp E, Harder T, Kohlhoff R, Dudenhausen JW, Plagemann A. Impact of breast-feeding on psychomotor and neuropsychological development in children of diabetic mothers: role of the late neonatal period. J Perinat Med2006;34(6):490-6.
17. von Beckerath A-K, Kollmann M, Rotky-Fast C, Karpf E, Lang U, Klaritsch P. Perinatal complications and long-term neurodevelopmental outcome of infants with intrauterine growth restriction. Am J Obstet Gynecol2013;208(2):130. e1-. e6.
18. Stehbens JA, Baker GL, Kitchell M. Outcome at ages 1, 3, and 5 years of children born to diabetic women. Am J Obstet Gynecol1977 Feb 15;127(4):408-13.
19. Auerbach J, Lerner Y, Barasch M, Palti H. Maternal and environmental characteristics as predictors of child behavior problems and cognitive competence. Amer J Orthopsychiat1992;62(3):409-20.
20. De Bie HMA, Oostrom KJ, Delemarre-van de Waal HA. Brain Development, Intelligence and Cognitive Outcome in Children Born Small for Gestational Age. Horm Res Paediatr2010;73(1):6-14.
21. Crawford SG, Kaplan BJ, Field LL. Absence of an association between insulin-dependent diabetes mellitus and developmental learning difficulties. Hereditas1995;122(1):73-8.
22. Hinkle SN, Schieve LA, Stein AD, Swan DW, Ramakrishnan U, Sharma AJ. Associations between maternal prepregnancy body mass index and child neurodevelopment at 2 years of age. Int J Obes (Lond)2012 Oct;36(10):1312-9.
23. Gage SH, Lawlor DA, Tilling K, Fraser A. Associations of maternal weight gain in pregnancy with offspring cognition in childhood and adolescence: findings from the Avon Longitudinal Study of Parents and Children. Am J Epidemiol2013 Mar 1;177(5):402-10.
24. Veldwijk J, Scholtens S, Hornstra G, Bemelmans WJ. Body mass index and cognitive ability of young children. Obes Facts2011;4(4):264-9.
25. Paterson J, Iusitini L, Gao W. Child developmental assessment at two-years of age: data from the Pacific Islands Families Study. Pac Health Dialog2011 Sep;17(2):51-63.
26. Meyer-Bahlburg HF, Dolezal C, Haggerty R, Silverman M, New MI. Cognitive outcome of offspring from dexamethasone-treated pregnancies at risk for congenital adrenal hyperplasia due to 21-hydroxylase deficiency. Eur J Endocrinol2012 Jul;167(1):103-10.
27. Riggins T, Bauer PJ, Georgieff MK, Nelson CA. Declarative memory performance in infants of diabetic mothers. Adv Child Dev Behav2011;38:73-110.
28. McCarthy AM, Lindgren S, Mengeling MA, Tsalikian E, Engvall JC. Effects of Diabetes on Learning in Children. Pediatrics2002 January 1, 2002;109(1):e9.
29. Jaddoe VV, Duijn C, Heijden A, Mackenbach J, Moll Ht, Steegers EP, et al. The Generation R Study: design and cohort update until the age of 4Â years. European Journal of Epidemiology2008;23(12):801-11.

30. Ornoy A. The impact of intrauterine exposure versus postnatal environment in neurodevelopmental toxicity: long-term neurobehavioral studies in children at risk for developmental disorders. Toxicol Lett2003 Apr 11;140-141:171-81.
31. Tanda R, Salsberry PJ, Reagan PB, Fang MZ. The impact of prepregnancy obesity on children's cognitive test scores. Matern Child Health J2013 Feb;17(2):222-9.
32. Brion MJ, Zeegers M, Jaddoe V, Verhulst F, Tiemeier H, Lawlor DA, et al. Intrauterine effects of maternal prepregnancy overweight on child cognition and behavior in 2 cohorts. Pediatrics2011 Jan;127(1):e202-11.
33. Basatemur E, Gardiner J, Williams C, Melhuish E, Barnes J, Sutcliffe A. Maternal prepregnancy BMI and child cognition: a longitudinal cohort study. Pediatrics2013 Jan;131(1):56-63.
34. Neggers YH, Goldenberg RL, Ramey SL, Cliver SP. Maternal prepregnancy body mass index and psychomotor development in children. Acta Obstet Gynecol Scand2003 Mar;82(3):235-40.
35. Casas M, Chatzi L, Carsin AE, Amiano P, Guxens M, Kogevinas M, et al. Maternal pre-pregnancy overweight and obesity, and child neuropsychological development: two Southern European birth cohort studies. Int J Epidemiol2013 Apr;42(2):506-17.
36. Pella D OK, Singh RB. Metabolic Syndrome: A Disease of the Brain. The Open Nutraceuticals Journal2011;4:107-18.
37. Van Lieshout RJ, Taylor VH, Boyle MH. Pre-pregnancy and pregnancy obesity and neurodevelopmental outcomes in offspring: a systematic review. Obes Rev2011 May;12(5):e548-59.
38. Winkley K, Ismail K, Landau S, Eisler I. Psychological interventions to improve glycaemic control in patients with type 1 diabetes: systematic review and meta-analysis of randomised controlled trials. BMJ2006 Jul 8;333(7558):65.
39. Nyaradi A, Li J, Hickling S, Foster J, Oddy WH. The role of nutrition in children's neurocognitive development, from pregnancy through childhood. Front Hum Neurosci2013;7:97.
40. Veena SR, Krishnaveni GV, Srinivasan K, Kurpad AV, Muthayya S, Hill JC, et al. Childhood cognitive ability: relationship to gestational diabetes mellitus in India. Diabetologia2010 Oct;53(10):2134-8.
41. Brand PLP, Molenaar NLD, Kaaijk C, Wierenga WS. Neurodevelopmental outcome of hypoglycaemia in healthy, large for gestational age, term newborns. Arch Dis Child2005 January 1, 2005;90(1):78-81.
42. Xu S, Yu B, Zepei J, Chang H, Guo J, Li B, et al. School performance affects adolescent blood pressure. Cardiol Young2013;124(3):459-63.
43. Nold JL, Georgieff MK. Infants of diabetic mothers. Pediatr Clin North Am2004;51(3):619-37.
44. Gazzolo D, Scopesi F, Russo A, Camoriano R, Santi F, Di Renzo GC, et al. Doppler velocimetry and behavioural state development in relation to perinatal outcome in pregnancies complicated by gestational diabetes. Early Hum Dev1995;41(3):193-201.
45. Aberg A, Westbom L. Association between maternal pre-existing or gestational diabetes and health problems in children. Acta Paediatr2001 Jul;90(7):746-50.
46. Kurjak A, Talic A, Honemeyer U, Stanojevic M, Zalud I. Comparison between antenatal neurodevelopmental test and fetal Doppler in the assessment of fetal well being. J Perinat Med2013 Jan;41(1):107-14.
47. Martinez-Frias ML, Bermejo E, Rodriguez-Pinilla E, Prieto L, Frias JL. Epidemiological analysis of outcomes of pregnancy in gestational diabetic mothers. Am J Med Genet1998 Jun 30;78(2):140-5.

48. Jeffery AN, Voss LD, Metcalf BS, Wilkin TJ. The impact of pregnancy weight and glucose on the metabolic health of mother and child in the south west of the UK. Midwifery2004;20(3):281-9.
49. Wu CS, Nohr EA, Bech BH, Vestergaard M, Olsen Jr. Long-Term Health Outcomes in Children Born to Mothers with Diabetes: A Population-Based Cohort Study. PLoS ONE2012;7(5):e36727.
50. Ostlund I, Hanson U, Bjorklund A, Hjertberg R, Eva N, Nordlander E, et al. Maternal and Fetal Outcomes if Gestational Impaired Glucose Tolerance Is Not Treated. Diabetes Care2003 July 1, 2003;26(7):2107-11.
51. Rijpert M, Evers IM, de Vroede MAMJ, de Valk HW, Heijnen CJ, Visser GHA. Risk Factors for Childhood Overweight in Offspring of Type 1 Diabetic Women With Adequate Glycemic Control During Pregnancy: Nationwide follow-up study in the Netherlands. Diabetes Care2009 November 1, 2009;32(11):2099-104.
52. Bhate V, Deshpande S, Bhat D, Joshi N, Ladkat R, Watve S, et al. Vitamin B12 status of pregnant Indian women and cognitive function in their 9-year-old children. Food Nutr Bull2008 Dec;29(4):249-54.
53. Hadden DR, Byrne E, Trotter I, Harley JMG, McClure G, McAuley RR. Physical and psychological health of children of Type 1 (insulin-dependent) diabetic mothers. Diabetologia1984;26(4):250-4.
54. Dionne G, Boivin M, Seguin JR, Perusse D, Tremblay RE. Gestational diabetes hinders language development in offspring. Pediatrics2007 Nov;122(5):e1073-9.
55. Rizzo TA, Silverman BL, Metzger BE, Cho NH. Behavioral adjustment in children of diabetic mothers. Acta Paediatr1997 Sep;86(9):969-74.
56. Dahlquist G, Kallen B. School marks for Swedish children whose mothers had diabetes during pregnancy: a population-based study. Diabetologia2007 Sep;50(9):1826-31.
57. Kowalczyk M, Ircha G, Zawodniak-Szalapska M, Cypryk K, Wilczynski J. Psychomotor development in the children of mothers with type 1 diabetes mellitus or gestational diabetes mellitus. J Pediatr Endocrinol Metab2002 Mar;15(3):277-81.
58. Bonilla C, Lawlor DA, Ben-Shlomo Y, Ness AR, Gunnell D, Ring SM, et al. Maternal and offspring fasting glucose and type 2 diabetes-associated genetic variants and cognitive function at age 8: a Mendelian randomization study in the Avon Longitudinal Study of Parents and Children. BMC Med Genet2012;13:90.
59. Siddappa AM, Georgieff MK, Wewerka S, Worwa C, Nelson CA, Deregnier RA. Iron deficiency alters auditory recognition memory in newborn infants of diabetic mothers. Pediatr Res2004 Jun;55(6):1034-41.
60. Silverman BL, Rizzo T, Green OC, Cho NH, Winter RJ, Ogata ES, et al. Long-term prospective evaluation of offspring of diabetic mothers. Diabetes1991 Dec;40 Suppl 2:121-5.
61. Torabi F, Sedigheh Amir Ali A, Saba A, Farin S, Hamid Alavi M. Correlation between high-risk pregnancy and developmental delay in children aged 4-60 months. Libyan J Med2012;7:18811.
62. Crowther CA, Hiller JE, Moss JR, McPhee AJ, Jeffries WS, Robinson JS. Effect of Treatment of Gestational Diabetes Mellitus on Pregnancy Outcomes. N Engl J Med2005 2014/01/20;352(24):2477-86.
63. Plagemann A, Harder T, Kohlhoff R, Rohde W, Dorner G. Glucose tolerance and insulin secretion in children of mothers with pregestational IDDM or gestational diabetes. Diabetologia1997;40(9):1094-100.
64. Buinauskiene JB, E.; Marmiene, V. The influence of glycemic control during pregnancy on the development of offspring of diabetic mothers. Acta medica lituanica2003;10(4):203-6.

65. Pintiaux A, Foidart J. Le diabete gestationnel. Rev Med Liege2005;60(5-6):338-43.
66. Mensah FK, Kiernan KE. Maternal general health and children's cognitive development and behaviour in the early years: findings from the Millennium Cohort Study. Child Care Health Dev2010;37(1):44-54.
67. Athanasiadis AP, Mikos T, Tambakoudis GP, Theodoridis TD, Papastergiou M, Assimakopoulos E, et al. Neurodevelopmental fetal assessment using KANET scoring system in low and high risk pregnancies. J Matern Fetal Neonatal Med 2013;26(4):363-8.
68. deRegnier R-A. Neurophysiologic evaluation of early cognitive development in high-risk infants and toddlers. Ment Retard Dev D R2005;11(4):317-24.
69. Weintrob N, Karp M, Hod M. Short- and long-range complications in offspring of diabetic mothers. J Diabetes Complications1996;10(5):294-301.
70. Malcolm J. Through the looking glass: gestational diabetes as a predictor of maternal and offspring long-term health. Diabetes Metab Res Rev2012;28(4):307-11.
71. Stenninger E, Flink R, Eriksson B, Sahlèn C. Long term neurological dysfunction and neonatal hypoglycaemia after diabetic pregnancy. Arch Dis Child Fetal Neonatal Ed1998 November 1, 1998;79(3):F174-F9.
72. Clausen T, Mortensen E, Schmidt L, Mathiesen E, Hansen T, Jensen D, et al. Cognitive deficits in adult offspring of women with gestational diabetes reflect confounding2012.
73. Torres-Española FJ, Jerez-Calero A, Segura MT, Moreno-Torres R, Robles C, Pérez-García M, et al. Neurodevelopmental status in babies born to overweight, obese or diabetic pregnant women, during the first 18th months of life2013.
74. Temple R, Hardiman M, Martinez-Cengotitabengoa MT, Alloway TP. Working memory in offspring of mothers with type 1 diabetes2010.
75. Rodriguez A, Miettunen J, Henriksen TB, Olsen J, Obel C, Taanila A, et al. Maternal adiposity prior to pregnancy is associated with ADHD symptoms in offspring: evidence from three prospective pregnancy cohorts. Int J Obes (Lond)2008 Mar;32(3):550-7.
76. Rodriguez A. Maternal pre-pregnancy obesity and risk for inattention and negative emotionality in children. J Child Psychol Psychiatry2010 Feb;51(2):134-43.
77. Baker BL, Neece CL, Fenning RM, Crnic KA, Blacher J. Mental Disorders in Five-Year-Old Children With or Without Developmental Delay: Focus on ADHD. Journal of Clinical Child & Adolescent Psychology2010 2014/01/20;39(4):492-505.
78. Lyall K, Pauls DL, Spiegelman D, Ascherio A, Santangelo SL. Pregnancy complications and obstetric suboptimality in association with autism spectrum disorders in children of the nurses' health study II. Autism Research2012;5(1):21-30.
79. Van Lieshout RJ, Voruganti LP. Diabetes mellitus during pregnancy and increased risk of schizophrenia in offspring: a review of the evidence and putative mechanisms. J Psychiatry Neurosci2008 Sep;33(5):395-404.
80. Rizzo TA, Metzger BE, Dooley SL, Cho NH. Early malnutrition and child neurobehavioral development: insights from the study of children of diabetic mothers. Child Dev1997 Feb;68(1):26-38.
81. Cummins M, Norrish M. Follow-up of children of diabetic mothers. Arch Dis Child1980 Apr;55(4):259-64.
82. Persson B, Gentz J. Follow-up of children of insulin-dependent and gestational diabetic mothers. Neuropsychological outcome. Acta Paediatr Scand1984 May;73(3):349-58.

83. Silverman BL, Rizzo TA, Cho NH, Metzger BE. Long-term effects of the intrauterine environment. The Northwestern University Diabetes in Pregnancy Center. Diabetes Care1998 Aug;21 Suppl 2:B142-9.
84. Rizzo TA, Dooley SL, Metzger BE, Cho NH, Ogata ES, Silverman BL. Prenatal and perinatal influences on long-term psychomotor development in offspring of diabetic mothers. Am J Obstet Gynecol1995 Dec;173(6):1753-8.
85. Temple RC, Hardiman M, Pellegrini M, Horrocks L, Martinez-Cengotitabengoa MT. Cognitive function in 6- to 12-year-old offspring of women with Type 1 diabetes. Diabet Med2011 Jul;28(7):845-8.
86. Plagemann A, Harder T, Kohlhoff R, Fahrenkrog S, Rodekamp E, Franke K, et al. Impact of early neonatal breast-feeding on psychomotor and neuropsychological development in children of diabetic mothers. Diabetes Care2005 Mar;28(3):573-8.
87. Chen Z, Zhao Y, Yang Y, Li Z. Leptin withdrawal after birth: a neglected factor account for cognitive deficit in offspring of GDM mother. Med Hypotheses2011 Jul;77(1):125-7.
88. Krakowiak P, Walker CK, Bremer AA, Baker AS, Ozonoff S, Hansen RL, et al. Maternal metabolic conditions and risk for autism and other neurodevelopmental disorders. Pediatrics2012 May;129(5):e1121-8.
89. Petersen MB, Pedersen SA, Greisen G, Pedersen JF, Molsted-Pedersen L. Early growth delay in diabetic pregnancy: relation to psychomotor development at age 4. Br Med J (Clin Res Ed)1988 Feb 27;296(6622):598-600.
90. Ratzon N, Greenbaum C, Dulitzky M, Ornoy A. Comparison of the motor development of school-age children born to mothers with and without diabetes mellitus. Phys Occup Ther Pediatr2000;20(1):43-57.
91. Ornoy A, Ratzon N, Greenbaum C, Wolf A, Dulitzky M. School-age children born to diabetic mothers and to mothers with gestational diabetes exhibit a high rate of inattention and fine and gross motor impairment. J Pediatr Endocrinol Metab2001;14 Suppl 1:681-9.
92. Ornoy A, Wolf A, Ratzon N, Greenbaum C, Dulitzky M. Neurodevelopmental outcome at early school age of children born to mothers with gestational diabetes. Arch Dis Child Fetal Neonatal Ed1999 Jul;81(1):F10-4.
